# Microbial Community Structure and Metabolic Function in the Venom Glands of the Predatory Stink Bug, *Picromerus lewisi* (Hemiptera: Pentatomidae)

**DOI:** 10.3390/insects15090727

**Published:** 2024-09-21

**Authors:** Jinmeng Li, Xu Tian, Tom Hsiang, Yuting Yang, Caihua Shi, Hancheng Wang, Wenhong Li

**Affiliations:** 1 College of Agriculture, Yangtze University, Jingzhou 434025, China; 2022710842@yangtzeu.edu.cn (J.L.); 2022720891@yangtzeu.edu.cn (X.T.); 519060@yangtzeu.edu.cn (Y.Y.); 2 Guizhou Academy of Tobacco Science, Guiyang 550081, China; 3 Institute of Plant Protection, Guizhou Academy of Agricultural Sciences, Guiyang 550006, China; 4 School of Environmental Sciences, University of Guelph, 50 Stone Road East, Guelph, ON N1G 2W1, Canada; thsiang@uoguelph.ca; 5 Institute of Advanced Agricultural Science, Hubei University of Arts and Science, Xiangyang 441053, China; shicaihua1980@126.com

**Keywords:** *Picromerus lewisi*, venom gland, microorganism, carbon source metabolism, high-throughput sequencing

## Abstract

**Simple Summary:**

*Picromerus lewisi* (*P. lewisi*), a predatory stink bug, is widely acknowledged as a beneficial natural enemy for controlling Lepidopteran pests in agroforestry systems. This species paralyzes its prey and facilitates extra-oral digestion by releasing venom. Although these insects harbor symbiotic microorganisms that play crucial roles in various biological aspects of their host interaction, comprehensive knowledge regarding the microbiota within the venom glands of *P. lewisi* is lacking. This study explored the numerous bacterial and fungal species in the venom glands of *P. lewisi* and the capability of these microbes to metabolize carbon sources. The dominant bacterial genera included *Wolbachia*, *Enterococcus*, *Serratia*, and *Lactococcus*, while the chief fungal genera were *Vishniacozyma*, *Cladosporium*, *Papiliotrema*, *Penicillium*, *Fusarium*, and *Aspergillus*. En masse, the microbial community showed abilities to metabolize a variety of carbon sources. This is the first report of the analysis of the microbiome in the venom glands of *P. lewisi*, enhancing our understanding of the microbial composition of this natural enemy’s venom. These findings will provide useful insights for researchers to further investigate the venom microbiota and their associated functionalities among predatory stink bugs.

**Abstract:**

The predatory stink bug, *Picromerus lewisi* (Hemiptera: Pentatomidae), is an important and valuable natural enemy of insect pests in their ecosystems. While insects are known to harbor symbiotic microorganisms, and these microbial symbionts play a crucial role in various aspects of the host’s biology, there is a paucity of knowledge regarding the microbiota present in the venom glands of *P. lewisi*. This study investigated the venom glands of adult bugs using both traditional in vitro isolation and cultural methods, as well as Illumina high-throughput sequencing technology. Additionally, the carbon metabolism of the venom gland’s microorganisms was analyzed using Biolog ECO metabolic phenotyping technology. The results showed 10 different culturable bacteria where the dominant ones were *Enterococcus* spp. and *Lactococcus lactis*. With high-throughput sequencing, the main bacterial phyla in the microbial community of the venom glands of *P. lewisi* were Proteobacteria (78.1%) and Firmicutes (20.3%), with the dominant bacterial genera being *Wolbachia*, *Enterococcus*, *Serratia*, and *Lactococcus*. At the fungal community level, Ascomycota accounted for the largest proportion (64.1%), followed by Basidiomycota (27.6%), with *Vishniacozyma*, *Cladosporium*, *Papiliotrema*, *Penicillium*, *Fusarium*, and *Aspergillus* as the most highly represented fungal genera. The bacterial and fungal community structure of the venom glands of *P. lewisi* exhibited high species richness and diversity, along with a strong metabolism of 22 carbon sources. Functional prediction indicated that the primary dominant function of *P. lewisi* venom-gland bacteria was metabolism. The dominant eco-functional groups of the fungal community included undefined saprotroph, fungal parasite–undefined saprotroph, unassigned, endophyte–plant pathogen, plant pathogen–soil saprotroph–wood saprotroph, animal pathogen–endophyte–plant pathogen–wood saprotroph, plant pathogen, and animal pathogen–endophyte–epiphyte–plant pathogen–undefined saprotroph. These results provide a comprehensive characterization of the venom-gland microbiota of *P. lewisi* and demonstrate the stability (over one week) of the microbial community within the venom glands. This study represents the first report on the characterization of microbial composition from the venom glands of captive-reared *P. lewisi* individuals. The insights gained from this study are invaluable for future investigations into *P. lewisi*’s development and the possible interactions between *P. lewisi*’s microbiota and some Lepidopteran pests.

## 1. Introduction

With the development of sustainable agriculture, the integrated management of pests is now viewed as an even more critical approach, and the use of natural enemies for the biological control of crop pests is one of the important links [1]. Many natural enemies including predatory and parasitic insects, as well as insect pathogens, have been studied for their potential in pest control [2,3]. Predatory insects and parasitoids serve as natural biological controllers of most insect pests in crop ecosystems [4,5]. There are approximately more than 20,000 species of predatory stink bugs [6]. They typically pounce on their prey by inserting needlelike probes and releasing toxic saliva to paralyze and digest it [7,8,9,10]. Predatory stink bugs are widely used in China to control aphids, thrips, and some Lepidopteran pests in fields and greenhouses [11,12,13,14]. Currently, very few species can be commercially produced, and the only common one is *Orius sauteri* (Hemiptera: Anthocoridae). There is a need to develop more species of predatory stinkbugs suitable for large-scale rearing. Since 2018, an increasing number of scholars have been investigating the biological characteristics of *Picromerus lewisi* (*P. lewisi*) by rearing them indoors [15]. These studies have revealed that the species is highly adaptable, exhibits significant egg production, and can be scaled up effectively for indoor rearing, demonstrating substantial potential for commercial rearing.

**Figure 1:**
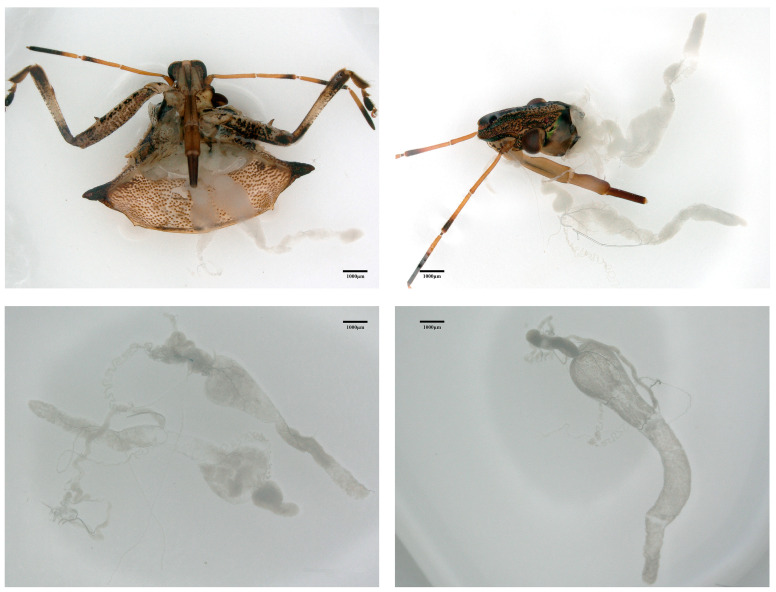
The venom glands of *Picromerus lewisi*.

*Picromerus lewisi* belongs to the subfamily of beneficial stink bugs (Asopinae) in the family of Pentatomidae (Hemiptera) and is mainly distributed in east Asia, as well as other Asian regions [16]. *P. lewisi* possesses a significant predation capacity, a broad predation range, and a formidable attacking force, particularly against key crop pests such as *Spodoptera frugiperda*, *Mythimna separata*, and *Ostrinia furnacalis*, as well as other Lepidopteran pests [17,18,19]. These pests can severely damage the leaves, fruits, or roots of crops, leading to substantial economic losses due to stunted growth, drastic reductions in yields, and, in some cases, even crop failure. Consequently, the predatory potential of *P. lewisi* on Lepidopteran pests establishes it as an important and valuable natural enemy in the ecosystem. The venom glands of *P. lewisi* are a complex organ with paired structures (Figure 1), subdivided into two anterior main glands (AMGs), two posterior main glands (PMGs), and two accessory glands (AGs) [20]. Furthermore, *P. lewisi* secretes venom from its poison glands, which is then stored, and the toxic saliva is transported from the venom glands through the ducts of the primary glands to the head, up to the mouthpart stylets, where it is injected into the prey [21]. There is evidence that predatory stink bugs’ venom consists primarily of organic and inorganic compounds with a high protein content. This venom induces intense pain and tissue damage to deter other predators. Alternatively, venom is used to rapidly paralyze and kill prey [22,23,24]. However, few studies are focusing on the components and functions of the venom of *P. lewisi*. Only one study identified the abundance of active neuropeptides in the venom of *P. lewisi* [25], and there are no previous reports on the presence of microorganisms in the venom or venom glands (also known as salivary glands) of *P. lewisi*.

The microbial communities of many insects play an important role in their physiological, metabolic, and immune functions. Symbiotic bacteria and fungi inhabit the intestinal tracts of host insects, potentially contributing essential nutrients to promote host growth, defend against predators, or enhance adaptation to the environment [26,27,28]. Surprisingly, in addition to the extensive microbial diversity present in the intestinal tissue, the salivary glands within the insect’s digestive system also contain microbial flora that play a vital part in the transmission of pathogens [29]. Although the microbial community in insect salivary glands may be of potential importance to their hosts, there is a scarcity of data regarding the complexity of this microbiota community. Moreover, the naturally occurring interactions between *P. lewisi* and microorganisms in the venom microenvironment remain largely unknown. To the best of our knowledge, the microbiological communities in the venom glands of *P. lewisi* have never been investigated. Venom glands serve as the primary organs of predation and defense for *P. lewisi*, and the microorganisms within them may be closely linked to their predatory abilities and defense mechanisms. There are several ways in which bacteria may influence predation or venom production [30]. In other well-studied cases, the microbiota plays a crucial role in regulating the host’s biochemical processes. For example, symbiotic bacteria can affect the host’s chemical processes by secreting bioactive secondary metabolites [31]. By isolating and identifying these microorganisms, we may uncover new bioactive compounds that could serve as the basis for innovative biopesticides in pest control. An increasing number of scholars are investigating microbial biocontrol resources to manage pests more effectively. For example, Tomar et al. [32] claimed that the nematodes along with their endosymbiotic bacteria had a biocontrol potential which could be used to reduce chemical pesticides, and the nematode–bacterium complexes were effective against a huge range of bacteria, fungi, nematodes and insects that are harmful to the crops. Therefore, conducting microbial research on insect venom glands to develop and utilize new microbial pest control technologies holds significant practical importance. This study aimed to explore the microbial community structure in the venom glands of *P. lewisi* and the ability of the microorganisms to metabolize carbon sources. Microorganisms from the venom glands were isolated and purified through in vitro culture cultivation. Additionally, Illumina high-throughput sequencing technology was employed to analyze the microbiological composition of the venom glands. Moreover, the Biolog ECO metabolic profiling technique was used to elucidate the metabolic roles of the microorganisms in the venom glands. Although insect microbial communities have been extensively studied, this research represents the first investigation specifically focusing on the microbial communities in the venom glands of *P. lewisi*. In this study, we not only examine the diversity of microorganisms present in the venom glands but also explore their capacity to metabolize various carbon sources. This exploration may reveal the potential roles these microorganisms play in host physiological functions and ecological adaptations. Furthermore, our research aims to enhance the current understanding of microorganisms in insect venom glands and offer new insights into their application in biological control.

## 2. Materials and Methods

### 2.1. Insects and Samples

The *P. lewisi* samples used in this study were collected from the Qianxinan natural-enemy breeding base in Guizhou Province. They were reared on the insect *Mythimna separata* as artificial feed in an artificial climate chamber. Male and female adults of *P. lewisi* were selected under controlled environmental conditions (27 ± 1 °C, 75% RH, and 16 h light/8 h dark photoperiod) for the tests.

### 2.2. Anatomy of the Venom Glands

Three hundred healthy adults of *P. lewisi* were randomly collected and divided into five groups each with 60 individuals, numbered Ag1, Ag2, Ag3, Ag4, and Ag5. They were dissected aseptically on a clean bench for venom-gland collection. The samples were washed with 75% ethanol for 2 min followed by 3% NaOCl (60 s), thoroughly rinsed with sterilized water to remove the disinfectant, air dried, and used for dissection.

Venom glands were collected following the method described by Su et al. [33] as follows. Adults were collected and transferred into sterile culture dishes, the legs were clipped with scissors, and the venom glands were removed intact by tearing away the body wall from the scutellum of the pronotum. The dissected venom glands were promptly transferred into 1.5 mL centrifuge tubes filled with sterile-filtered phosphate-buffered saline (pH = 7.4) and stored in a freezer at −80 °C for high-throughput sequencing. In addition, a portion of venom-gland samples was collected for microbial isolation and culture, followed by a Biolog-ECO analysis. The sample numbers of the venom glands of *P. lewisi* were Ag1, Ag2, Ag3, Ag4, and Ag5. The tweezers were sanitized before each use throughout the experiment to ensure that all extracts originated from the venom glands of *P. lewisi*.

### 2.3. Isolation of Culturable Microorganisms from the Venom Glands of P. lewisi

The adult samples of *P. lewisi* were selected to isolate microorganisms from the venom glands as follows. A total of 60 adults from the population were selected. The samples were surface-disinfected with 75% ethanol for 2 min followed by 3% NaOCl for 60 s, thoroughly rinsed with sterilized water to remove the disinfectant, and the venom gland contents of *P. lewisi* were then isolated and homogenized with 1 mL of PBS (pH = 7.4). Portions (0.1 mL of each venom-gland suspension (diluted 10-fold) were transferred to 2 mL centrifuge tubes containing sterile saline and subsequently diluted to 10^−9^. Aliquots of 100 µL of the 10^−5^ to the 10^−8^ venom-gland dilutions were spread onto the surface of plates of each bacterial medium (Luria–Bertani and nutrient agar) and each fungal medium (potato dextrose agar, alkyl ester agar, and oatmeal agar). Following inoculation, the plates were incubated at 30 °C in the dark for 48 h. Bacteria and fungi with diverse morphological traits (e.g., colors, growth rates, and morphologies) were selected from the media, and a single representative isolate of each morphotype was then transferred to a new plate of NA for bacteria and PDA for fungi. After three to four successive passages, the purified strains were stored for an extended period at −20 °C in a solution containing 15% glycerol.

### 2.4. Molecular Characterization

The bacteria were revived on NA plates before use, with a single colony selected for re-suspension in 20 µL of lysis buffer, mixed well, and used as a DNA template [34].

The 16S rRNA gene of each bacterium was amplified via PCR using the forward primer 27F (5′-AGAGTTTGATCCTGGCTCAG-3′) and reverse primer 1492R (5′-GGTTACCTTGTTACGACTT-3′). The PCR amplifications were performed in a thermocycler (Veriti™ 96 Well Thermal Cycler; Applied Biosystems, CA, USA) in a 30 µL reaction system that contained 2 µL of DNA template, 1.5 µL of each primer, 15 µL of Taq DNA polymerase (EX Taq version 2.0 Plus Dye), and 10 µL of ddH_2_O. PCR conditions were as follows: denaturation at 98 °C for 10 s, annealing at 50 °C for 30 s, and extension at 72 °C for 30 s, ending with a final extension at 72 °C for 5 min. The PCR products were visualized using 1.0% Biowest Agarose gel electrophoresis and sequenced by Shanghai Sangon Biotech Company (Shanghai, China). The nucleotide sequences obtained were submitted to the NCBI (https://www.ncbi.nlm.nih.gov) database, and their accession numbers are available in GenBank.

### 2.5. DNA Extraction, PCR Amplification, and Illumina NovaSeq PE250 Sequencing

Total bacterial and fungal DNA from the venom glands of *P. lewisi* was extracted using the CTAB technique. The DNA purity and concentration were assessed on 2% agarose gels, and subsequently diluted to the appropriate concentration (1 ng/μL). DNA samples were stored at −20 °C before being used for PCR. For the microbial diversity analysis, diluted genomic DNA was used as a template for the PCR amplification of the V3 to V4 regions of the 16S rDNA genes using primers 341F (5′-GGACTACNNGGGTATCTAAT-3′) and 806R (5′-GGACTACNNGGGGTATCTAAT-3’), and of the ITS1 region using primers ITS5-1737F (5′-GGAAGTAAAAGTCGTAACAAGG-3′) and ITS2-2043R (5′-GCTGCGTTCTTCATCGATGC-3′) [35,36]. The PCR was performed using the following cycle: 98 °C for 1 min, followed by 30 cycles of 98 °C for 10 s, 50 °C for 30 s, and 72 °C for 30 s, and a final extension at 72 °C for 5 min. Amplicons were extracted from 2% agarose gels, followed by magnetic bead purification for PCR products that met quality standards, and purified PCR products were then quantified via an enzymatic assay. After purification, the recovered products were used to construct bacterial 16S rDNA and fungal ITS high-throughput sequencing libraries using the DNA library prep kit. Lastly, the libraries were sequenced using the NovaSeq 6000 platform (Illumina company, San Diego, CA, USA) with 250 bp paired-end reads. All sequencing and basis analysis was performed by Novogene (Tianjin, China).

### 2.6. Sequence Processing and Microbial Diversity Analysis

The data for each sample were segregated from the downlinked data based on barcode sequences and PCR amplification primer sequences. Subsequently, the reads of each sample were spliced using FLASH (version 1.2.11) to obtain the raw reads. The raw reads were then strictly filtered and processed using FASTP (v0.23.1). The sequences were then cross-referenced with the database to identify and eliminate chimeric sequences, resulting in the acquisition of the final valid dataset. The data were analyzed using QIIME2 (v202006), first by reducing noise using the DADA2 module in QIIME2 to obtain the ASVs (amplicon sequence variants) and feature tables. The 16S rRNA and ITS sequences were annotated using the Silva database and Unite database libraries, respectively (with a threshold of 0.8~1) [37,38]. Finally, the data were normalized to the smallest sample. The alpha diversity index of bacterial and fungal communities in the samples was computed using QIIME2 and visualized using R software (v4.0.3). Furthermore, the ASV abundance table was standardized using the PICRUSt software (v1.1.4), and the functional prediction of the bacterial community was conducted by matching the Greengene ID of each ASV with the KEGG database [39]. FUNGuild function prediction was performed using Python (v2.7.15), where species were compared to the FUNGuild database to obtain the corresponding trophic type for the ASV. Absolute and relative abundances for each trophic type were calculated using an unhomogenized abundance table, and the relative abundances were used to complete the subsequent statistical tests and visualizations [40].

### 2.7. Metabolic Functional Analysis of Carbon Sources in Microbial Communities

Sixty venom glands were dissected aseptically and placed into a sterile centrifuge tube with 1 mL of 0.9% sterile saline, immediately ground to homogenize, and fixed to 15 mL with 0.9% sterile saline. Lastly, 120 μL of the sample was added to the Biolog ECO microplate using an 8-channel micropipette. Subsequently, the inoculated ECO plate was placed in the OmniLog system and incubated at 28 °C. The OmniLog system automatically reads and analyzes the data once the continuous reading has reached a plateau. The size of the OmniLog reading reflects the strength of the metabolic capability of the microbial community. Biolog D5EOKA_data.exe software (v1.7) was used to collect color changes in the metabolic pores of toxic gland microorganisms during their growth process. The darker the color of the metabolic pore, the larger the OmniLog reading, and the stronger the metabolic ability.

### 2.8. Statistics Analysis

Flower diagrams were created using the SVG function in Perl to visualize species overlap among different samples. Species abundance was statistically analyzed by identifying the top 10 to 30 species based on the abundance in each sample across various taxonomic levels (phylum, order, family, genus, and species). Histograms depicting the distribution of relative abundance were generated using the SVG function in Perl. To evaluate the metabolism of 31 carbon sources by the venom-gland microorganisms of *P. lewisi*, we created and analyzed heat maps using Hemi software (v1.0). Data collation and visualization were conducted using Excel 2019 and Origin 2021. Image processing was carried out with Adobe Photoshop CS6 to ensure the clarity and accuracy of graphical representations.

## 3. Results

### 3.1. Culturable Microorganisms from the Venom Glands of P. lewisi

**Figure 2:**
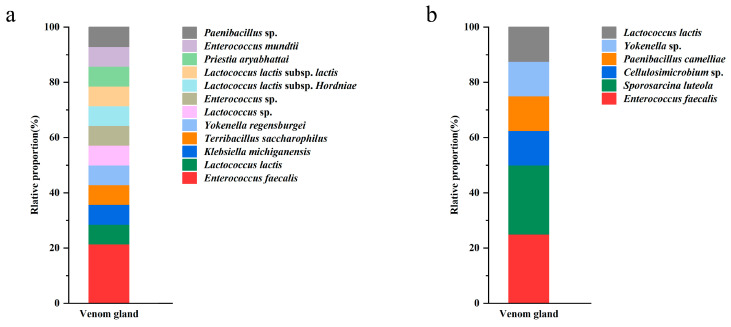
Frequency of isolation of bacteria from venom glands of *P. lewisi* on NA (**a**) and LB (**b**) media.

The 22 morphotypes of bacterial strains were subjected to 16S sequencing which revealed that they belonged to 2 phyla, 6 families, 9 genera, and 10 unique species. *Enterococcus* spp. was represented by seven isolates, with the highest separation frequency (Figure 2). *Lactococcus lactis* was represented by five isolates, *Yokenella* spp., *Paenibacillus* spp., and *Sporosarcina luteola* were represented by two isolates, while the rest of the taxa only had single representatives (Table 1). No fungi were isolated from the samples.

### 3.2. Sequencing Data Statistics and Analysis

**Figure 3:**
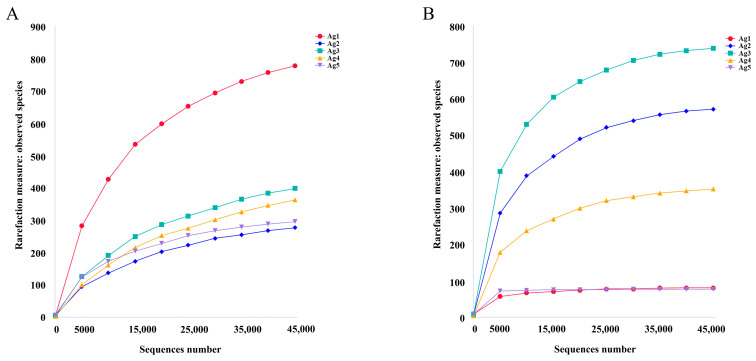
Rarefaction curves based on 16S rDNA sequencing of bacterial (**A**) and fungal (**B**) communities.

From 16S sequencing, 605,328 high-quality reads were obtained from the five samples. The average sequence length was 427 bp. From ITS sequencing, 414,238 high-quality sequences were obtained among 106,933,791 bases and an average sequence length of 256 bp. The rarefaction curves indicated that the sequencing depth was adequate to reliably estimate the diversity of microorganisms present in all samples (Figure 3).

### 3.3. Microbial Community in the Venom Glands of P. lewisi

**Figure 4:**
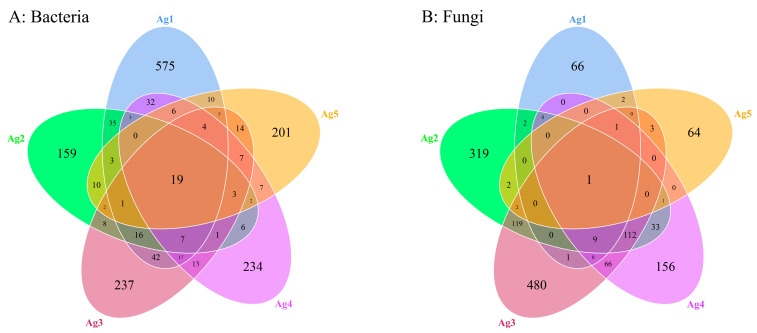
Venn analysis of shared and unique ASVs of bacteria (**A**) and fungi (**B**) among five venom-gland samples of *P. lewisi*.

A total of 1679 bacterial ASVs and 1439 fungal ASVs were identified within the microbiota of the venom gland. The Ag1–Ag5 samples exhibited 575, 159, 237, 234, and 201 unique bacterial ASVs, as well as 66, 319, 480, 156, and 64 unique fungal ASVs, respectively (Figure 4). The results of the alpha diversity analysis (Table 2) showed that the average Shannon index of bacterial and fungal communities was 2.03 and 3.96, and additionally, the Simpson index was 0.48 and 0.78. The coverage was above 0.997, indicating that the sequencing results were representative, and these results may truly reflect the diversity of bacteria and fungi in the venom glands of *P. lewisi*.

**Figure 5:**
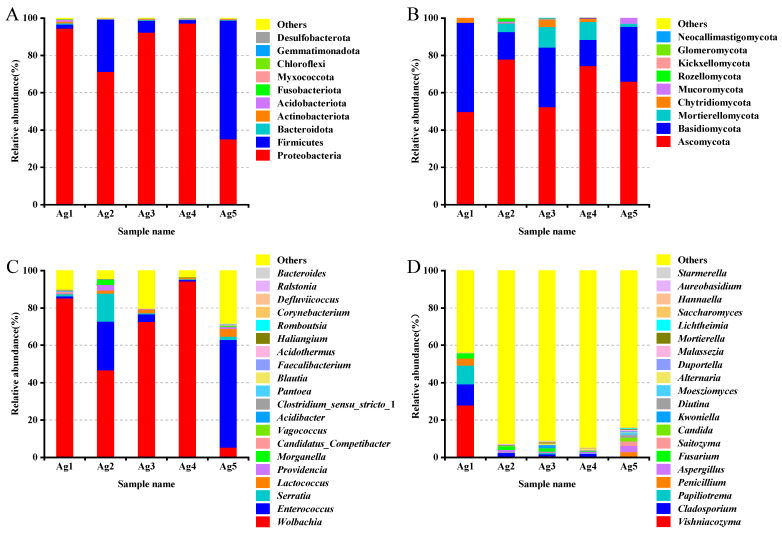
Community composition of the bacteria ((**A**) phylum, (**C**) genus) and fungi ((**B**) phylum, (**D**) genus) in the venom glands of *P. lewisi*.

Based on the relative abundance analyses across all samples, the microbial community composition at the phylum level in venom-gland samples showed that the 16S rDNA sequences were predominantly classified as Proteobacteria (78.1%), Firmicutes (20.3%), Actinobacteriota (0.4%), Acidobacteriota (0.3%), Bacteroidota (0.3%), Myxococcota (0.2%), Chloroflexi (0.1%), Gemmatimonadota (0.1%), Desulfobacterota (<0.1%), and Verrucomicrobiota (<0.1%). Among them, Proteobacteria and Firmicutes were the dominant phyla (Figure 5A). The proportion of unknown reads ranged from 42% to 95% among the five samples. A closer inspection of these reads using a BLAST analysis of a subsample of 100 against the NCBI database revealed that most of the “unknown” reads matched *Cuminum*, plant species whose DNA was also amplified by the ITS primers. These were eliminated from the overall results, leaving only fungal taxa. At the fungal community level, Ascomycota accounted for the largest proportion (64.1%), followed by Basidiomycota fungi (27.6%) (Figure 5B).

For bacterial reads, at the class level, Alphaproteobacteria was the dominant class among the bacterial samples, accounting for an average relative abundance of 61.5%, followed by Bacilli (19.9%) and Gammaproteobacteria (16.6%). Ten classes were annotated for venom gland fungi, including Tremellomycetes, Sordariomycetes, Dothideomycetes, Eurotiomycetes, Agaricomycetes, and Saccharomycetes, with relative abundances between 1 and 10% each. At the order level, the bacteria of the venom glands of P. lewisi were distributed in 10 orders, including Rickettsiales, Lactobacillales, Enterobacterales, Burkholderiales, Lachnospirales, Bacteroidales, Pseudomonadales, Corynebacteriales, Bifidobacteriales, and Propionibacteriales. Among them, the relative abundance of Rickettsiales, Lactobacillales, and Enterobacterales was higher, accounting for 60.9%, 19.9%, and 15.6%, respectively, and the remaining seven orders accounted for less than 1%. The dominant fungal orders of venom glands were Tremellales, Hypocreales, Capnodiales, Eurotiales, Pleosporales, and Saccharomycetales, with relative abundances of 9.3%, 6.3%, 3.2%, 2.8%, 2.0%, and 1.2%, respectively (Table 3).

At the genus level, the most abundant bacterial genera were *Wolbachia* (60.8%), *Enterococcus* (17.9%), *Serratia* (3.8%), and *Lactococcus* (1.8%) (Figure 5C). In the first four genera analyzed, two belonged to the phylum Proteobacteria and two to the phylum Firmicutes. The fungi were mainly distributed in 20 genera including *Vishniacozyma*, *Cladosporium*, *Papiliotrema*, *Penicillium*, *Fusarium*, *Aspergillus*, *Saitozyma*, and *Candida*. Among them, *Vishniacozyma* was the primary genus of fungi with a relative abundance of 5.9%, followed by *Cladosporium*, *Papiliotrema*, *Penicillium*, *Fusarium*, *Aspergillus*, with abundances of 3.2%, 2.3%, 1.4%, 1.3%, and 1.3%, respectively (Figure 5D).

### 3.4. PICRUSt and FUNGuild Functional Prediction Analysis

**Figure 6:**
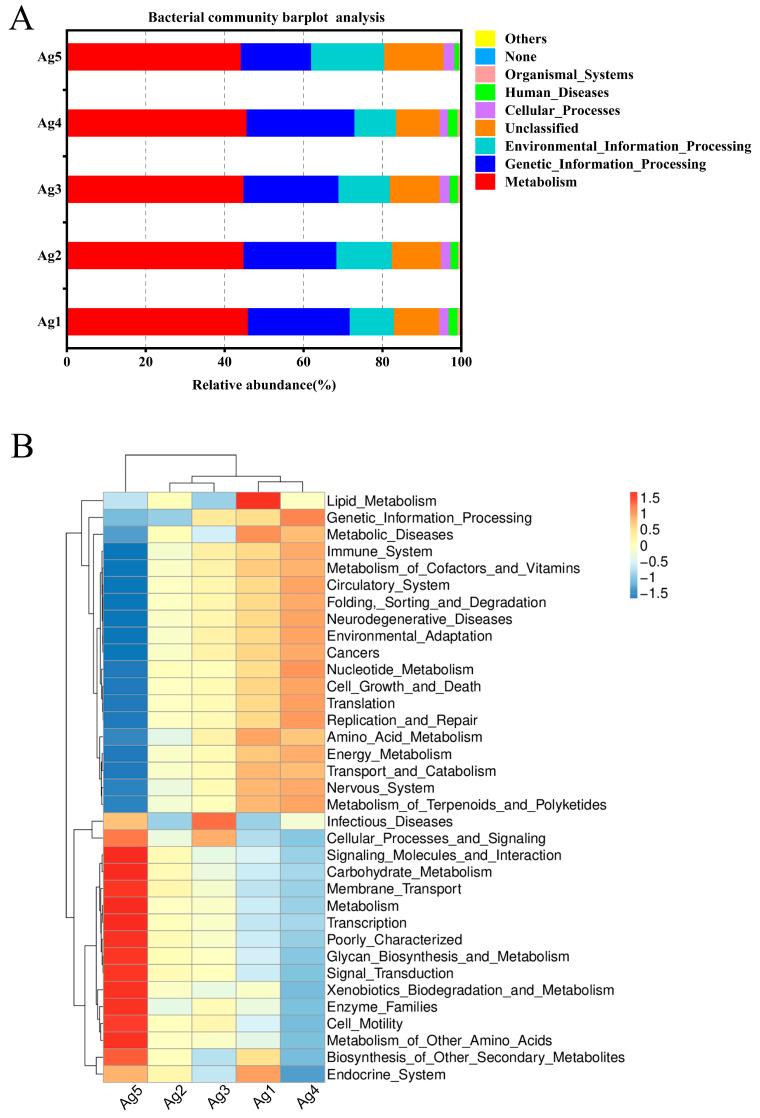

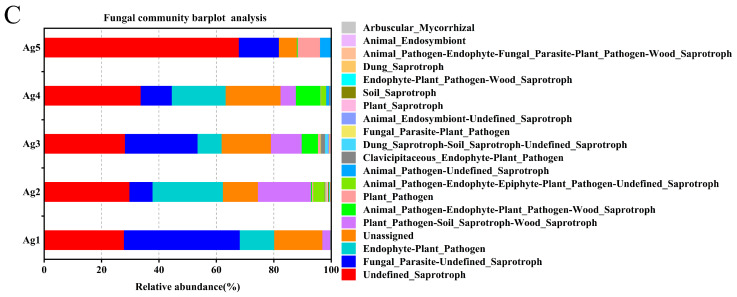
A histogram and heatmap showing the hierarchical classification of the predicted KEGG orthologs’ functional profiles (KEGG levels 1 and 2) across all samples of the bacterial microbiota (**A**,**B**). FUNGuild prediction of the relative abundance of fungal communities in the venom glands of *P. lewisi* (**C**).

To gain a better understanding of the metabolic functions of *P. lewisi*’s venom-gland microbiota, the potential roles of bacteria and fungi were predicted using PICRUSt (v1.1. 4) and FUNGuild software (Python 2.7.15). All predicted KEGG metabolic pathways were categorized at the first and second hierarchical levels. At the primary functional level, the dominant functional group of *P. lewisi* venom-gland bacteria was metabolism (45.1%), which accounted for nearly half of the abundance of all the primary pathways (Figure 6A). This result also showed that the venom-gland bacteria of *P. lewisi* mainly performed the function of metabolizing various substances. At the secondary functional level, the bacterial community exhibited the highest relative abundance in membrane transport. However, the analysis of the five samples revealed that these communities were consistently enriched in genes associated with replication and repair, carbohydrate metabolism, amino acid metabolism, translation, and energy metabolism (Figure 6B).

FUNGuild was used to predict fungal communities’ functional groups from five samples. The results revealed that the endophytic fungi in the venom glands of *P. lewisi* had four dominant ecological functional groups, among which parasites, saprotrophs, pathotrophs, and pathotroph–saprotrophs were the major components. The eight major functional subtypes were undefined saprotroph, fungal parasite–undefined saprotroph, unassigned, endophyte–plant pathogen, plant pathogen–soil saprotroph–wood saprotroph, animal pathogen–endophyte–plant pathogen–wood saprotroph, plant pathogen, and animal pathogen–endophyte–epiphyte–plant pathogen–undefined saprotroph, with average abundance percentages of 37.43%, 19.67%, 14.27%, 12.69%, 7.44%, 2.99%, 1.87%, and 1.17%, respectively (Figure 6C).

### 3.5. Metabolic Activities of Microbial Communities in the Venom Glands of P. lewisi towards Diverse Carbon Sources

**Figure 7:**
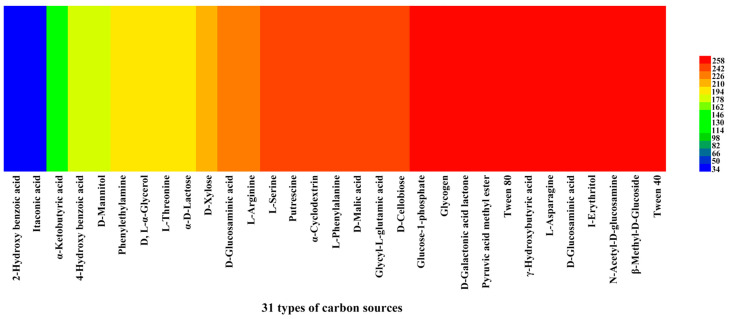
Heat map of 31 carbon sources’ metabolism abundance of the venom glands of *P. lewisi*. Note: Colors range from blue to shades of green and red to indicate low, medium, and high carbon source utilization, respectively, assessed as arbitrary OmniLog values.

We investigated the reading of venom-gland samples on the ECO plates was investigated between 0 and 168 h. The results showed that the venom-gland samples of *P. lewisi* could metabolize 29 carbon sources normally, among which 22 carbon sources including α-cyclodextrin, glycogen, L-arginine, L-asparagine, I-erythritol, putrescine, D-galactonic acid lactone, and other carbon sources were metabolized with high efficiency. The lowest metabolic utilization was γ-hydroxybutyric acid, followed by 2-hydroxy benzoic acid and itaconic acid, which could not be metabolized (Table 4, Figure 7).

## 4. Discussion

In addition to the midgut, the salivary glands of insects also play a pivotal role in replicating and transmitting pathogens [41,42,43]. For example, in the salivary glands of insects such as fruit flies, mosquitoes, and leafhoppers, the presence of bacteria is considered to be a way to spread to plants or other intermediate hosts [44,45]. While there has been extensive research on microbial diversity in insect hosts, the focus has primarily been on the insect gut [46,47,48]. There have been few studies on the microbial diversity of insect salivary glands. Herein, we investigated for the first time the diversity of endophytic microorganisms in the venom glands (also known as salivary glands) of *P. lewisi* as well as the metabolic functions of the venom-gland microorganisms in response to carbon sources in five samples.

In this study, we isolated and identified the microorganisms in the venom glands of *P. lewisi*, and we observed that the bacteria present could be relatively easily isolated and cultured, while the culturable fungi were not observed. Ten distinct bacterial species classified under the phyla Proteobacteria and Firmicutes were recognized. *Enterococcus* and *Lactococcus* were identified as the predominant bacterial species among the microbial population. It has been reported that Pseudomonas is the dominant microbiota in *Anopheles* mosquitoes’ salivary glands. Additionally, six different species of *Saccharomyces* were also discovered in the salivary glands [49]. Herein, the ten bacteria found in the venom glands of *P. lewisi* may imply that these bacteria are capable of overcoming the barrier and adapting to the special microenvironment of venom glands to live. Moreover, research has indicated that *Pseudomonas aeruginosa* and *Enterobacter* sp. dominate the bacterial community in the salivary glands of the cicada *Hyalessa maculaticollis* (Motschulsky) (Hemiptera: Cicadidae) [50]. The findings exhibit notable disparities in the types of cultivable microorganisms present in the venom glands of *P. lewisi*, which could be attributed to ecological and dietary distinctions between the two insects.

In the high-throughput sequencing data analysis, the predominant bacterial phylum in the venom glands of *P. lewisi* was Proteobacteria, followed by Firmicutes, while other phyla made up a tiny percentage. Similarly, Xue et al. [51] and An et al. [52] reported that the phyla Proteobacteria and Firmicutes were the predominant organisms in the microbiomes of both *Adelphocoris suturalis* and *Apolygus lucorum*. It is well known that microorganisms of the phylum Proteobacteria and Firmicutes are commonly found in the insect epidermis and intestinal tract [53,54]. Both harmful and beneficial microorganisms play a vital role in providing nutrients for the host’s healthy growth, with their effects largely influenced by the host’s diet. Our study found that *Wolbachia* was abundant in the venom glands of *P. lewisi*, followed by *Enterococcus*, *Serratia*, and *Lactococcus*. Previous research has shown that over 65% of insect species harbor *Wolbachia*, an intracellular symbiotic bacterium commonly found in arthropods [55]. Ashraf et al. [56] found through a comparative microbiome analysis that *Wolbachia* was the dominant endosymbiotic bacterium in *Diaphorina citri* and its associated parasitoids *Tamarixia radiata* and *Diaphorencyrtus aligarhensis*. It can not only cause cytoplasmic incompatibility, parthenogenesis, feminization, and male-killing [57], but it has also been found to have significant inhibitory effects on mosquito-borne viruses and parasites. It can also protect the host by activating a mix of drug resistance and disease tolerance mechanisms, and these actions are amphoteric [58]. *Enterococcus* was the second most dominant genus in the venom glands of *P. lewisi*. Hanchi et al. [59] claimed that *Enterococcus* has the potential to act as a probiotic, with the gastrointestinal tract of animals being considered the primary reservoir. However, Esmaeilishirazifard et al. [60] found that the presence of Enterococcus faecalis was also found in the venom glands of snakes, indicating its wide ecological adaptability. Moreover, *Enterococcus faecalis* has the capability to assist the host in diminishing the insecticidal effects of *Bacillus thuringiensis*, thus helping the gypsy moth to cope with complex environments [61]. Recent research has found that *Enterococcus* sp. can trigger immune signaling pathways and modulate infection [62], and *Enterococcus* peptidoglycan can promote checkpoint inhibitor cancer immunotherapy [63]. Ishak et al. [64] reported the presence of *Lactococcus lactis* in *Solenopsis invicta*. It is a fermenting bacterium known to produce lactic acid from sugar and antimicrobial substances, and it may play an important role in the digestive system of ant larvae. A recent study revealed the abundant presence of *Lactococcus lactis* in *Apolygus lucorum* at various developmental stages [65], although its specific function remains unreported. This finding is in line with our research results. The presence of bacteria in the venom gland further confirms their adaptability to extreme environments. However, not all bacteria are beneficial; some are classified as opportunistic pathogens. For instance, *Serratia marcescens* has been isolated from insects like honey bees and *Plutella xylostella* [66,67]. In some cases, the strains can be highly virulent and kill the larvae within 2–3 days, with symptoms similar to a viral infection [68,69]. Our functional prediction results also provided an alternative explanation: most bacterial functions were focused on “metabolism”, “genetic information processing”, and “environmental information processing” to support insect growth and development and to enable adaptation to the environment. Further studies might include sequencing the genomes of bacterial symbionts to delve more comprehensively into these metabolic functions.

Within the fungal communities of the venom-gland samples, *Vishniacozyma*, *Cladosporium*, *Papiliotrema*, *Penicillium*, *Fusarium*, and *Aspergillus* were identified as the dominant taxa. Previous studies have shown that *Vishniacozyma* has the potential to biocontrol yeast and inhibit fungal infections caused by *Penicillium expansum*, *Botrytis cinerea*, and *Cladosporium* sp. [70,71]. Fungi of the genus *Cladosporium* (Dothideomycetes) are widely distributed. Studies on insect-associated *Cladosporium* have shown that *Cladosporium* is prevalent in studies of Hemiptera and Coleoptera, reflecting their importance in agricultural environments [72]. In a similar study concerning the congeneric Queensland fruit fly (*Bactrocera tryoni*), *Cladosporium* was the dominant fungi in the gut mycobiome, with a higher frequency in females [73]. Additionally, *Cladosporium* is the most common fungus associated with arthropods, and there is increasing evidence that *Cladosporium* may also infect insects and cause epidemics within pest populations or facilitate plant defense responses. Nymphs and adults of the sugarcane white wooly aphid (*Ceratovacuna lanigera*: Hemiptera, Aphididae) were completely overgrown by *Cladosporium cladosporioides* mycelium, which penetrated and disrupted their powdery waxy coating [74]. Therefore, we can use these microbial resources to control agricultural and forestry pests more efficiently. There may be differences between the microbes in the venom glands of *P. lewisi* and those in other parts of the insect, such as the gut or body surface. We speculate that these differences are due to the varying environmental and functional requirements of microbes in different parts of the insect [75].

In this work, we used PICRUSt to anticipate the potential function of *P. lewisi*’s venom-gland bacteria. The results showed that all samples exhibited a high metabolic capacity. This implies that venom-gland bacteria have the ability to metabolize a variety of substances. Through FUNGuild prediction, we discovered that venom-gland fungi may play a role in decomposing, parasitizing, and causing plant and animal diseases, which could have significant implications for their health and growth. This implied an intricate association between these fungi and other organisms, and concurrently, it exhibits the diversity, complexity, and potential ecological functions of venom-gland microorganisms within the ecosystem. Nevertheless, further studies are needed for functional validation.

The results of traditional microbial isolation and culture were inconsistent with the results of high-throughput sequencing. *Serratia* sp., as well as the above-mentioned fungi, were not isolated from the venom glands of *P. lewisi*. This points out the limitation of traditional microbial isolation and culture methods where microbial communities may not be fully represented, and where nutrient and temperature requirements may restrict the types and numbers of cultural microbiota observed. The use of high-throughput sequencing methods to analyze both the bacterial and fungal components of the microbiome avoids the well-known difficulties of isolating microorganisms by traditional culture-dependent methods. Mathew et al. [76] found that *Bacillus* could facilitate termite growth through interacting with fungi, e.g., the conjunction of Bacillus with *Termitomyces* may be conducive to the decomposition of lignin in the termite gut. However, a predictive analysis of the interactions between bacteria and fungi in the venom glands of *P. lewisi* was not performed in this study, and further research is needed. In order to understand the carbon-source metabolism characteristics of microbial communities in the venom glands of *P. lewisi*, analyzing the metabolic functions of microbial communities is essential, which is also meaningful for carrying out functional studies of the microorganisms in the venom glands.

In our study, the venom-gland microbial community was able to metabolize 29 carbon sources, among which 22 carbon sources had the highest utilization efficiency, while 2-hydroxybenzoic acid and itaconic acid were hardly metabolized. The results were different from those reported in previous studies [77]. This phenomenon may be attributed to the acidic environment of the venom glands of *P. lewisi*, which restricts the utilization of certain carbon sources by specific microorganisms due to their high specificity. During growth, microorganisms produce a variety of enzymes, including cellulases, amylases, and esterases. The inability of microorganisms to metabolize particular carbon sources suggests a deficiency in the corresponding enzymes, which may hinder their survival and reproduction in specific environments, ultimately affecting their competitiveness within their ecological niche. We focused on studying the microbial community structure and metabolism of carbon sources in the venom glands, but we were not sure what functions these microbes performed in the glands. It is well known that the venom of *P. lewisi* is rich in proteins, including venom hemolysin-like, venom protein family, and serpin [21]. We speculate that these microbial communities may influence the composition of the venom as well as the genetic structure of the venom glands, which will be crucial for our understanding of the evolution of venom and antibiotic resistance [78,79]. Research into the microbiome of venom is an emerging field. The study of venom microbes and the correlation of microbial community profiles with functional characteristics of venom microbial communities will deepen our understanding of the mechanisms that drive venom variation [80]. Therefore, we will focus on the specific functions of these microorganisms in the venom glands of *P. lewisi* in the future. Follow-up studies will further investigate the impact of microbial communities on venom composition and function using genomics and transcriptomics. Therefore, we will conduct further investigations into the specific functions of these microorganisms in the venom glands of *P. lewisi*. Additionally, we will explore the effects of microbial communities on the predation and venom production of *P. lewisi* using genomics and transcriptomics approaches in future studies.

## 5. Conclusions

This study used an Illumina sequencing system to characterize the venom-gland microbiota of *P. lewisi* throughout its adult stage. As a result, the microbial community in the venom glands of *P. lewisi* was characterized by high metabolic adaptability and diversity. The venom glands of *P. lewisi* contained bacterial and fungal species. The dominant bacteria were *Wolbachia*, *Enterococcus*, *Serratia*, and *Lactococcus* sp., and the primary fungi were *Vishniacozyma*, *Cladosporium*, *Papiliotrema*, *Penicillium*, *Fusarium*, and *Aspergillus* sp., with a strong metabolizing ability of carbon sources. The study improves the knowledge of the venom-gland microbiota in *P. lewisi* at the species level. The results presented in this study offer valuable insights for prospective research on the microbiota of predatory stink bugs.

## Figures and Tables

**Table 1:** Bacteria isolates from the venom glands of *P. lewisi*.

Strain Designation	Closest NCBI Match	Accession Number	Similarity (%)
X1	*Klebsiella michiganensis*	OR037498	99.67
X2	*Terribacillus saccharophilus*	OR037499	99.47
X3	*Lactococcus lactis*	OR037500	99.05
X7	*Yokenella regensburgei*	OR037501	99.25
X8	*Lactococcus lactis* subsp. *lactis*	OR037502	99.56
X10	*Yokenella* sp.	PP516327	97.52
X11	*Enterococcus* sp.	PP516328	96.53
X12	*Enterococcus faecalis*	PP5163299	98.45
X13	*Lactococcus lactis* subsp. hordniae	OR037503	99.67
X15	*Lactococcus* sp.	OR685638	99.47
X18	*Priestia aryabhattai*	OR037504	99.38
X25	*Lactococcus lactis*	OR685647	99.68
X29	*Paenibacillus* sp.	PP516330	97.63
X30	*Enterococcus faecalis*	PP516331	98.65
X31	*Sporosarcina luteola*	PP516332	98.60
X32	*Sporosarcina luteola*	PP516333	98.15
X33	*Cellulosimicrobium* sp.	PP516334	98.31
X34	*Enterococcus faecalis*	PP516335	98.86
X36	*Paenibacillus camelliae*	PP516336	99.78
X37	*Enterococcus faecalis*	PP516337	99.17
X38	*Enterococcus faecalis*	PP516338	98.69
X39	*Enterococcus mundtii*	PP516339	99.53

**Table 2:** Alpha diversity indices of bacteria and fungi in the venom glands of *P. lewisi*.

Sample Groups		Diversity Indices ^b^				
		Observed Species ^a^	Chao1	Shannon	Simpson	Coverage
Bacteria	Ag1	775	847.25	1.67	0.27	0.996
	Ag2	275	301.81	2.52	0.71	0.998
	Ag3	396	490.90	1.80	0.46	0.997
	Ag4	361	478.47	0.68	0.11	0.997
	Ag5	294	316.16	3.46	0.85	0.999
	Average	420.20	486.92	2.03	0.48	0.997
Fungi	Ag1	82	83.91	4.31	0.93	1.00
	Ag2	600	665.56	4.26	0.84	0.998
	Ag3	794	857.52	4.89	0.86	0.998
	Ag4	379	437.91	1.97	0.37	0.998
	Ag5	76	76.00	4.35	0.90	1.00
	Average	386.20	424.18	3.96	0.78	0.999

“^a^” Observed species was defined as the number of visually observed species (OTUs). “^b^” Chao1 diversity estimated the total number of species contained in the community sample, with higher values representing lower abundance. Shannon diversity was the total number of taxa in the samples and their proportion. Simpson diversity characterized the diversity and evenness of species distribution within the community. Coverage was the depth of the sequencing index.

**Table 3:** Relative abundance of the top 10 bacteria and fungi at the class and order levels in the venom glands of *P. lewisi*.

Bacteria ^b^		Fungi ^b^	
Class	Order	Class	Order
Alphaproteobacteria (61.5%)	Rickettsiales (60.9%)	Tremellomycetes (9.4%)	Tremellales (9.3%)
Bacilli (19.9%)	Lactobacillales (19.9%)	Sordariomycetes (6.5%)	Hypocreales (6.3%)
Gammaproteobacteria (16.6%)	Enterobacterales (15.6%)	Dothideomycetes (5.4%)	Capnodiales (3.2%)
Clostridia (0.4%)	Burkholderiales (0.4%)	Eurotiomycetes (2.8%)	Eurotiales (2.8%)
Bacteroidia (0.3%)	Lachnospirales (0.2%)	Agaricomycetes (1.7%)	Pleosporales (2.0%)
Actinobacteria (0.3%)	Bacteroidales (0.1%)	Saccharomycetes (1.2%)	Saccharomycetales (1.2%)
Acidobacteriae (0.2%)	Pseudomonadales (0.1%)	Mortierellomycetes (0.6%)	Mortierellales (0.6%)
Polyangia (0.1%)	Corynebacteriales (——)	Ustilaginomycetes (0.3%)	Ustilaginales (0.3%)
Thermoleophilia (0.1%)	Bifidobacteriales (——)	Malasseziomycetes (0.2%)	Malasseziales (0.2%)
Ktedonobacteria (——) ^a^	Propionibacteriales (——)	Mucoromycetes (0.1%)	Corticiales (0.1%)

“^a^” indicates the relative abundance of taxa was <0.1%. “^b^” Data were defined as the relative abundance of each taxon of the microbial community members for all samples.

**Table 4:** Metabolic capacity of the venom-gland microbial community of *P*. *lewisi* on 31 carbon sources.

Carbon Source Type	Substrates	Metabolic Activity ^a,b,c^
Carbohydrates	β-Methyl-D-Glucoside	+++ ^b^
	D-Xylose	++ ^c^
	α-Cyclodextrin	+++
	Glycogen	+++
	D-Cellobiose	+++
	Glucose-1-phosphate	+++
	α-D-Lactose	++
Amino acids	L-Arginine	+++
	L-Asparagine	+++
	L-Phenylalanine	+++
	L-Serine	+++
	L-Threonine	++
	Glycyl-L-glutamic acid	+++
Alcohols	I-Erythritol	+++
	D-Mannitol	++
	D, L-α-Glycerol	++
Amines	N-Acetyl-D-glucosamine	+++
	Phenylethylamine	++
	Putrescine	+++
Acids	D-Glucosaminic acid	+++
	2-Hydroxy benzoic acid	- ^a^
	4-Hydroxy benzoic acid	+++
	γ-Hydroxybutyric acid	+
	D-Galacturonic acid	+++
	Itaconic acid	-
	α-Ketobutyric acid	+++
	D-Malic acid	+++
Esters	D-Galactonic acid lactone	+++
	Pyruvic acid methyl ester	+++
	Tween 40	+++
	Tween 80	+++

“^a^” indicates no capacity to metabolize carbon sources. “^b^” indicates strong metabolism of carbon sources. “^c^” indicates the average metabolism of carbon sources.

## Data Availability

Data available on request, the raw reads of 16S and ITS sequencing data were deposited into the NCBI Sequence Read Archive database (accession nos.: PRJNA1134270 and PRJNA1134282).
